# ML Flow serological test: complementary tool in leprosy^⋆^

**DOI:** 10.1016/j.abd.2022.05.005

**Published:** 2023-03-06

**Authors:** Janaína Olher Martins Montanha, Susilene Maria Tonelli Nardi, Fernanda Modesto Tolentino Binhardi, Heloisa da Silveira Paro Pedro, Milena Polotto de Santi, Vânia Del Arco Paschoal

**Affiliations:** a Centro de Laboratório Regional de São José do Rio Preto, Instituto Adolfo Lutz, São José do Rio Preto, SP, Brazil; b Department of Nursing in Collective Health, São José do Rio Preto Faculty of Medicine, São José do Rio Preto, SP, Brazil

**Keywords:** Contact tracing, Leprosy, Public health, Serology, Surveillance

## Abstract

**Background:**

The evaluation of household contacts of leprosy cases allows the early diagnosis of new cases.

**Objective:**

To associate the results of the ML Flow test with the clinical characteristics of leprosy cases and to verify their positivity in household contacts, in addition to describing the epidemiological profile of both.

**Methods:**

Prospective study with patients diagnosed over the course of one year (n = 26), without prior treatment, and their household contacts (n = 44) in six municipalities in northwestern São Paulo, Brazil.

**Results:**

There was a predominance of men among the leprosy cases, of 61.5% (16/26); 77% (20/26) were over 35 years old; 86.4% (22/26) were multibacillary; 61.5% (16/26) had a positive bacilloscopy; and 65.4% (17/26) had no physical disability. The ML Flow test was positive in 53.8% (14/26) of the leprosy cases and was associated with those who had a positive bacilloscopy and were diagnosed as multibacillary (p-value <0.05). Among the household contacts, 52.3% (23/44) were women and aged over 35 years; 81.8% (36/44) had been vaccinated with BCG ‒ Bacillus Calmette-Guérin. The ML Flow test was positive in 27.3% (12/44) of household contacts, all of whom lived with multibacillary cases; seven lived with positive bacilloscopy cases and six with consanguineous cases.

**Study limitations:**

Difficulty in convincing the contacts to undergo the evaluation and collection of the clinical sample.

**Conclusion:**

The ML Flow test, when positive in household contacts, can help the identification of cases that require more attention by the health team, as it indicates a predisposition to disease development, especially when they are household contacts of multibacillary cases, with positive bacilloscopy and consanguineous. The ML Flow test also helps in the correct clinical classification of the leprosy cases.

## Introduction

Brazil is one of the few countries worldwide not to have reached the leprosy elimination level, that is, a prevalence of less than one case/10,000 inhabitants.^1^ To achieve the goal of leprosy control set by the World Health Organization (WHO) and the Ministry of Health (MoH), one of the effective actions is contact surveillance, which aims at identifying new cases among those who live or have lived together on a prolonged basis, with the newly diagnosed case of leprosy.^2^

People who live and have contact with patients are exposed to a 3.8-fold greater risk of becoming ill when compared to the general population^3^, ^4^ and household contacts of multibacillary (MB) cases represent a group characterized by high exposure to the bacillus.^5^ Therefore, from an epidemiological point of view, one of the key activities to prevent increased transmission of leprosy is the examination of contacts, which could increase the earlier diagnosis and cure rates of the disease.^4^

Clinical dermato-neurological examination and positive bacilloscopy are still considered superior for defining the diagnosis of leprosy. Although bacilloscopy helps in the diagnosis of the disease, it has low sensitivity, especially in the paucibacillary (PB) forms, and may also be negative in some MB patients due to inadequate procedures for collection, staining, reading and the possibility that the bacillus is not present in the collection sites.^6^, ^7^

The serodiagnosis expands the list of laboratory tests that collaborate in the diagnosis of leprosy. Phenolic glycolipid-1 (PGL-I) antigen is the main antigenic glycolipid of *Mycobacterium leprae* producing antibodies of the IgG (immunoglobulin type G) and IgM (immunoglobulin type M) classes, related to disease activity and clinical form.^8^, ^9^ The detection of IgM antibodies to phenolic glycolipid I (PGL-I) in serodiagnosis is the best standardized test for leprosy.

ML Flow is a quick and easy-to-perform test for the detection of M-immunoglobulin antibodies to *M. leprae* PGL-I. Like other serological tests, ML Flow is not considered reference for the diagnosis but can be used as a complementary tool for clinical classification after the initial diagnosis,^10^ definition and monitoring of drug therapy, evaluation of recurrence, and in the selection of contacts who are at most risk of becoming ill.^11^, ^12^, ^13^

This study aimed to associate the results of the ML Flow test in the index case with their clinical classification, to verify the frequency of positive ML Flow results in the household contacts and to delineate the epidemiological profile of the cases and their household contacts.

## Methods

The study was carried out in six municipalities in the northwest region of the state of São Paulo, which will be designated as municipalities I, II, III, IV, V and VI, with a total population of 574,151 inhabitants. Among the selected municipalities, five of them have a prevalence considered to be medium to very high by the Ministry of Health (1.0 to 19.99 cases per 10,000 inhabitants)^14^ and are considered priority municipalities according to the list of the 2016 Leprosy Control Program.^15^

It included all treatment-naïve new cases diagnosed with leprosy throughout one year, from October 2018 to October 2019, and their household contacts.

Only treatment-naïve cases were selected since after treatment is started, the tendency is for a decrease in bacillary load and IgM class antibodies. Moreover, considering that the test chosen for the study was a qualitative one, this could interfere with its sensitivity.

To characterize the profile of the index case and their household contacts, an analysis of existing clinical records at the health units and an interview were carried out using specific forms for collecting sociodemographic data.

In the index cases, blood sample collection for the ML Flow test was performed at the time of the interview, after the patient signed the free and informed consent (FIC) form.

Household contacts were invited to participate by telephone, with prior scheduling of the location, date and time for collection of samples, at the convenience of the household contact, the service and the professional. In case of non-attendance, a new telephone call and scheduling were carried out, aiming to obtain all research data in the maximum period of 60 days after the diagnosis of the index case.

All household contacts underwent dermatological examinations and a Simplified Neurological Assessment (SNA). Blood collection for the ML Flow test was performed after authorization and signature of the FIC form.

After the blood sample collection, in both cases, the sample was sent to the Reference Laboratory, Centro Regional do Instituto Adolfo Lutz, located in São José do Rio Preto, along with the form that was filled out according to the previously established flow. The standard operating procedure of Instituto Adolfo Lutz was used for the collection and storage of biological samples. The procedure used to perform the ML Flow test, as well as the interpretation of the results, were as described in the test package insert by Buhrer-Sekula et al., 2003.^16^

The results were entered into an Excel spreadsheet and analyzed using the EPI INFO.7 statistical program developed by the Centers for Disease Control and Prevention (CDC). The frequency of the studied variables was verified and Fisher's Exact Test was used to confirm the association between the variables, with p ≤ 0.05 values being considered significant.

The project was approved by the Research Ethics Committee of Instituto Adolfo Lutz, under Counsel number 2,118,254.

## Results

During the study period, blood samples were collected from 26 new leprosy index cases in the following municipalities: one case in municipalities I and VI, five in municipality II and 19 cases in municipality V. Municipalities III and IV did not collect samples during the study period. These new cases originated from 61 household contacts, of which 44 (72.3%) were interviewed, in addition to being submitted to blood collection and dermato-neurological examinations. A total of 17 (27.7%) contacts were excluded: seven could not be located (incorrect address and telephone number available or had moved to another city), four refused to participate in the research, and six, despite four telephone contacts and two appointments, did not attend the appointment (Table 1).

**Table 1:** Frequency distribution of the sociodemographic data of leprosy cases and their household contacts.

Sociodemographic data		Cases (n = 26)		Household contacts (n = 44)	
		n	%	n	%
Sex	Male	16	61.5	21	47.7
	Female	10	38.5	23	52.3
Age	0 to 15	1	3.8	10	22.7
	16 to 35	5	19.2	11	25.0
	36 to 55	8	30.8	14	31.8
	>55	12	46.2	9	20.5
Level of schooling	Illiterate	1	3.8	1	2.3
	Incomplete Elementary School	17	65.5	18	40.9
	Complete Elementary School	3	11.5	5	11.4
	High School	4	15.4	14	31.8
	Higher Education	1	3.8	4	9.1
	Postgraduate Education	–	–	2	4.5
Marital status	Single	5	19.2	19	43.2
	Married	12	46.2	18	40.9
	Separated/Divorced	–	–	2	4.5
	Widowed	2	7.7	1	2.3
	Common-law marriage	7	26.9	4	9.1
Employment status	Employed	14	53.8	22	50.0
	Unemployed	6	23.1	17	38.6
	Retired/medical leave	6	23.1	5	11.4
Housing status	Owned house	17	65.4	37	84.1
	Rented house	9	34.6	7	15.9

The mean age was 49.3 years (SD = 18.3) for the index case and 40.4 years (SD = 29.4) for the household contacts. The average found was 2.35 (SD = 1.54) contacts for each case.

Regarding basic sanitation and piped water supply, 100% of the households were supplied by municipal network services.

Both in index cases (19/26) and in household contacts (25/44) there was a predominance of white individuals. In 50% (13/26) of the interviewed leprosy cases, the family income was up to two minimum wages.

A total of four people lived in 46.2% (12/26) of the households, and 59.1% had up to four rooms. The mean time the contacts had been living with the cases was 14 years (SD = 10.58), and 88.6% of the household contacts did not live with other cases of leprosy outside the home in the last ten years and had never lived with another person with the same disease (Table 2).

**Table 2:** Frequency distribution of the characteristics of household contacts and ML Flow test results.

Situation of contact		ML Flow of contacts				Total	p-value*
		Positive		Negative			
		n	%	n	%	n (%)	
Time living with the patient in the same house	Less than five years	5	11.4	6	13.6	11 (25.0)	0.46
	Five years or more	6	13.6	17	38.7	23 (52.3)	
	No information	1	2.3	9	20.4	10 (22.7)	
	Total	12	27.3	32	72.7	44 (100)	
Degree of kinship with the case	Consanguineous	6	13.6	14	31.8	20 (45.5)	0.48
	Non-consanguineous	6	13.6	18	40.9	24 (54.5)	
	Total	12	27.3	32	72.7	44 (100)	
Underwent neuro-dermatological examination	Yes	7	15.9	20	45.5	27 (61.4)	0.63
	No	3	6.8	9	20.4	12 (27.3)	
	Does not know	2	4.5	3	6.8	5 (11.4)	
	Total	12	27.3	32	40.9	44 (100)	
Received BCG vaccine	Yes	10	22.7	26	59.1	36 (81.8)	0.64
	No	1	2.3	2	4.5	3 (6.8)	
	No information	1	2.3	4	9.1	5 (11.4)	
	Total	12	27.3	32	40.9	44 (100)	

BCG, Bacillus Calmette-Guérin (BCG).

* Fisher’s exact test.

Dermato-neurological examinations were performed in 61.4% (27/44) of the household contacts. It was observed that, even among the contacts that had received the BCG vaccine, 22.7% (10/44) had a positive ML Flow test result (Table 3).

**Table 3:** Frequency distribution of sociodemographic data when related to the result of the ML Flow serological test in household contacts (n = 44).

Sociodemographic data		ML Flow contacts				Total	p-value^a^
		Positive		Negative			
		n	%	n	%	n (%)	
Gender	Male	5	11.3	16	36.4	21 (47.7)	0.44
	Female	7	15.9	16	36.4	23 (52.3)	
Age	0 to 15	2	4.6	8	18.2	10 (22.8)	0.55
	16 to 35	4	9.1	7	15.9	11 (25.0)	
	36 to 55	3	6.8	11	25.0	14 (31.8)	
	Over 55	3	6.8	6	13.6	9 (20.4)	
Level of schooling	Illiterate	1	2.3	‒	‒	1 (2.3)	0.09
	Incomplete Grade School	7	15.9	11	25.0	18 (40.9)	
	Complete Grade School	‒	‒	5	11.4	5 (11.4)	
	High School	3	6.8	11	25.0	14 (31.8)	
	Higher Education	1	2.3	3	6.8	4 (9.1)	
	Postgraduate Education	–	–	2	4.5	2 (4.5)	
Degree of kinship	Son/Daughter	2	4.5	10	22.7	12 (27.2)	0.48
	Spouse	4	9.1	11	25.0	15 (34.1)	
	Brother/Sister	3	6.8	1	2.3	4 (9.1)	
	Father/Mother	1	2.3	2	4.5	3 (6.8)	
	Brother/Sister-in-law	‒	‒	2	4.5	2 (4.5)	
	Stepson/daughter	‒	‒	4	9.1	4 (9.1)	
	Grandson/daughter	‒	‒	1	2.3	1 (2.3)	
	Daughter-in-law	1	2.3	1	2.3	2 (4.6)	
	Stepfather	1	2.3	‒	‒	1 (2.3)	

a Fishers' exact test.

The positive result for ML Flow in the contacts was more frequent in women 15.9% (7/44); aged over 35 years, 13.6% (6/44); and those who had incomplete elementary education or were illiterate, 18.2% (8/44).

Positive ML Flow test, when related to the degree of kinship of the contact and the case, was more frequent in the spouse (a) 4.9% (4/44), followed by siblings 6.8% (3/44) and children, 4.5% (2/44). When observing the total number of collections of each degree of kinship with positivity, the number of siblings stands out: four collections and, of these, three were positive (Table 4).

**Table 4:** Frequency distribution of clinical data related to the result of the ML Flow serological test in leprosy cases (n = 26).

Clinical data of cases		ML Flow cases				Total	p-value^a^
		Positive		Negative			
		n	%	n	%	n (%)	
Clinical form	Indeterminate	‒	‒	1	3.8	1 (3.8)	0.01
	Tuberculoid	‒	‒	3	11.5	3 (11.5)	
	Borderline	4	15.4	1	3.8	5 (19.2)	
	Lepromatous	10	38.5	7	27.0	16 (65.5)	
Bacilloscopy	Positive	12	46.1	4	15.4	16 (61.5)	0.00
	Negative	2	7.7	8	30.8	10 (38.5)	
Biopsy b	Suggestive of leprosy	5	41.7	5	41.7	10 (83.4)	0.31
	Negative	‒	‒	2	16.6	2 (16.6)	
Degree of disability	With DPD	5	19.2	4	15.4	9 (34.6)	0.61
	Without DPD	9	34.6	8	30.8	17 (65.4)	
Operational classification	Paucibacillary	‒	‒	5	19.2	5 (19.2)	0.01
	Multibacillary	14	53.8	7	27.0	21 (80.8)	

DPD, Degree of Physical Disability, according to the World Health Organization.

a Fishers' exact test.

b 14 cases had no information about the biopsy in their medical records.

Among cases diagnosed with borderline and lepromatous (MB) clinical forms, when the bacilloscopy was associated with the MF Flow test, the results were compatible in 20 (76.9%) cases, 12 (60%) of which were positive and 8 (40%) negative for both tests.

When comparing the result of the ML Flow test of the index case with its operational classification (PB or MB), it was observed that all patients who were classified as PB, that is, five (19.2%) had a negative result, whereas among MB patients 14 were positive (53.8%) and seven (27%) were negative.

The mean time of symptom onset for cases that had a positive serology, 53.8% (14/26) was 31.7 months (SD = 37.9) with a median of 12 months (min. six and max. 32 months). For cases that had a negative serology (n = 12) the mean was 38.75 months (SD = 33.54), with a median of 30 months (min. five and max. 99 months; Table 5).

**Table 5:** Results of ML Flow serological tests of leprosy cases and respective household contacts.

	Result of the ML Flow test of household contacts	
Result of the ML Flow test in leprosy cases	Positive (n = 12)	Negative (n = 32)
Positive	7 (15.9%)	17 (38.6%)
Negative	5 (11.4%)	15 (34.1%)
Total	12 (27.3%)	32 (72.7%)

## Discussion

Anti-PGL-I antibodies, mainly of the IgM class, are considered specific for *M. leprae* and are found in leprosy patients; however, they can also be found at low titers in exposed individuals. Antibodies do not confer protection and indicate *M. leprae* infection.^17^ ML Flow test positivity in contacts is an indirect indicator of the spread of *Mycobacterium leprae* infection in the general population.^18^

In this study, the finding of 27.3% of seropositivity in household contacts is consistent with the literature and demonstrated seropositivity for ML Flow between 15.6% in PB^19^, ^20^ to 28.6% in MB cases.^16^, ^18^ The monitoring of these contacts showed that those with a positive ML Flow test result have a greater risk of developing the MB forms than seronegative contacts, even without clinical evidence compatible with the disease, a fact that reaffirms the need for a more effective follow-up of this family group.^21^

Most seropositive contacts were female; however, to date, there is no consensus on this information in the literature. Some studies show greater seropositivity in males,^18^, ^22^ while others show it in females.^23^, ^24^, ^25^ It should be recalled that women seek health services more frequently than men, which should encourage greater access to health services and the diagnosis of leprosy.^23^ In the analysis, no association was found between sociodemographic data and ML Flow results in household contacts.

The highest positivity occurred among household contacts of MB index cases (borderline and lepromatous). These data, which corroborates existing studies,^16^, ^18^, ^20^, ^22^ is an expected finding since household contacts of patients with MB leprosy in the lepromatous form showed a 3.8-fold higher risk of developing leprosy than contacts of patients with other clinical forms.^3^

The mean time of symptom onset found in this study suggests the possibility that cases with a positive serology have less resistance to Hansen's bacillus; therefore, they become ill earlier than those with a negative serology.

The findings of this study regarding the age group and level of schooling also converge with those found in the literature, with a predominance of the age group older than 35 years and a low level of schooling (incomplete elementary school or illiterate).^18^, ^21^, ^26^ Low level of schooling can cause difficulty in understanding the information disclosed about leprosy and may favor the spread and risk of contracting the disease. Moreover, studies show an inverse relationship between the number of school years and the family income level, showing that most have low income and are unemployed or self-employed, suggesting this disease is associated with socioeconomic factors.^25^, ^26^

The occurrence of new cases among consanguineous household contacts of the index case, mainly first-degree relatives, was 2.05 times more likely than other types of kinship, demonstrating the importance of genetic susceptibility in the disease transmission chain, widely documented in the literature.^3^, ^27^, ^28^

The mean time of 14 years of contact with leprosy cases and the fact that most of these contacts had not lived, in the last ten years, with other people infected with the disease outside the household, confirm the understanding that to reach the elimination of the disease as a public health problem, it is necessary to monitor household contacts, which are people who live in the same house or who have continuous and prolonged contact with the patient,^29^ since this population is more susceptible to the disease.^25^

Although the dermato-neurological examination is one of the guidelines for the epidemiological investigation of household contacts described in the disease prevention and control manual, the examination was performed in only 61.4% of the contacts,^14^ indicating that the health service needs to focus attention in this issue. On the other hand, of the assessed contacts, most had at least one BCG vaccine scar, which constitutes a satisfactory finding for this group, since this vaccine was included as a leprosy control measure to be used in contacts with the different clinical forms of the disease.^14^

The index cases classified as MB (borderline and lepromatous) showed a positivity rate for the ML Flow test of 53.9%; while the PB cases showed no positive results, diverging from the sensitivity reported in the literature, where studies indicate that the sensitivity of some types of rapid tests (NDO-LID, NDO, LID or PGL-1) in MB patients varies from 80% to 95%, while in PB patients, when detectable, it is lower, and ranges from 15% to 64%.^17^, ^21^

The classification of leprosy based on the number of lesions in PB (≤5 dermatological lesions) and MB (>5 dermatological lesions) cases can lead to an inappropriate therapeutic regimen, resulting in the undertreatment of MB patients, when misclassified as PB. This therapeutic failure is the concern of health professionals who, without access to laboratory tests, tend to treat more patients as MB. In a study carried out in Nigeria, it was observed that 95.7% of the patients received multidrug therapy for MB, while only 62.9% had a positive ML Flow test result. Of these, 55.9% would have up to five skin lesions, and therefore would be classified as PB, as recommended by the WHO.^17^

All index cases also underwent bacilloscopy, and when the results of the ML Flow test and the bacilloscopy were compared, the agreement was 75%. However, 15.4% of the index cases had a positive result only in the bacilloscopy and 7.7% only in the ML Flow test. The negative results had a 100% agreement for both tests. These findings are in disagreement with those found in the literature, where the ML Flow test is reported to have a greater sensitivity than bacilloscopy,^17^, ^30^ but can be justified due to the constant collection training for bacilloscopy carried out in the studied region.

The clinical-histopathological agreement with the ML Flow test results found in the present study is in line with data found in the literature.^31^

A late diagnosis increases the risk of physical impairment caused by the silent progression of the bacillus action in the body, especially in peripheral nerves.^32^ Although the studied region is considered of low endemicity for leprosy, the findings of the present study indicate high rates of physical disability at the time of the diagnosis, which suggests late diagnosis.

No laboratory test alone is sufficiently sensitive and specific for the correct clinical classification in all forms of leprosy, but they are important auxiliary tools for obtaining a correct diagnosis.

ML Flow test is a quick, individual, easy-to-perform test, with sensitivity and specificity similar to that of ELISA,^17^ and generated a better cost/benefit ratio for the research. In addition, it does not require laboratory equipment to perform it, converging with the Brazilian Unified Health System (SUS – Sistema Único de Saúde) strategies. Its use in SUS was approved by Ordinance SCTIE/MoH n. 84, of December 31, 2021, and its distribution will be gradual and decentralized to the municipalities.^33^

Among the limiting factors for this study, the difficulty in locating and convincing contacts to come to the health services for evaluation and sample collection can be highlighted. Another difficulty was compiling the results of the laboratory tests, because, despite the existence of a network of reference laboratories with defined flows for the care of these patients, some municipalities send the bacilloscopy tests and biopsies to different laboratories, without any quality control.

## Conclusion

The authors conclude that the ML Flow test is an effective tool both to select household contacts who are predisposed to the development of the disease and need closer monitoring, and to help in the correct clinical classification of leprosy cases. Thus, the results suggest that the ML flow test can speed up diagnosis, helping to prevent the physical disabilities still observed in people affected by leprosy in this region.

According to the results of the present study, the contacts of multibacillary patients, with positive bacilloscopy and consanguineous relatives, seem to be more exposed to the leprosy bacillus. Low income, low level of schooling and unsatisfactory living conditions can be risk factors for contracting leprosy among household contacts, and the follow-up of these groups is necessary to interrupt disease transmission.

## Financial support

Fundação Paulista Contra Hanseníase – Project number 183.

## Authors' contributions

Janaína Olhar Martins Montanha: Participated in the design and planning of the study; collection, analysis and interpretation of results; writing and critical review of the manuscript.

Susilene Maria Tonelli Nardi: Participated in the design and planning of the study; collection, analysis and interpretation of results; writing and critical review of the manuscript; and research orientation.

Fernanda Modesto Tolentino Binhardi: Contributed to the design and planning of the study; writing and critical review of the manuscript.

Heloisa da Silveira Paro Pedro: Contributed to the design and planning of the study; writing and critical review of the manuscript.

Milena Polotto de Santi: Contributed to the design and planning of the study; writing and critical review of the manuscript.

Vânia Del Arco Paschoal: Participated in the design and planning of the study; collection, analysis and interpretation of results; writing and critical review of the manuscript; and research orientation.

## Conflicts of interest

None declared.
